# Effects of the first month of lockdown for COVID-19 in Italy: A preliminary analysis on the eyecare system from six centers

**DOI:** 10.1177/1120672120953074

**Published:** 2021-09

**Authors:** Roberto dell’Omo, Mariaelena Filippelli, Francesco Semeraro, Teresio Avitabile, Fabrizio Giansanti, Francesco Parmeggiani, Mario R Romano, Diego Strianese, Vito Romano, Gianni Virgili, Ciro Costagliola

**Affiliations:** 1 Department of Medicine and Health Sciences “Vincenzo Tiberio,” University of Molise, Campobasso, Italy; 2 Eye Clinic, Department of Neurological and Vision Sciences, University of Brescia, Brescia, Italy; 3 Department of Ophthalmology, University of Catania, Catania, Italy; 4 Department of Ophthalmology, University of Florence, Florence, Italy; 5 Department of Morphology, Surgery and Experimental Medicine, University of Ferrara, Ferrara, Italy; 6 Department of Ophthalmology, Humanitas Gavazzeni – Castelli, Bergamo, Italy; 7 Orbital Unit, CME Department, King Khalid Eye Specialist Hospital, Riyadh, Saudi Arabia; 8 Department of Eye and Vision Science, University of Liverpool, Liverpool, UK

**Keywords:** COVID-19, coronavirus outbreak, pars plana vitrectomy, phacoemulsification

## Abstract

**Purpose:**

To compare the number of eye surgical procedures performed in Italy during the first month of lockdown with those performed in the same period in 2019.

**Methods:**

Review of the surgical procedures performed from 10 March to 9 April 2019 and from 10 March to 9 April 2020 (the first month of lockdown because of the COVID-19 outbreak) at six academic institutional centers of Italy. A distinction was made between urgent procedures: any trauma repairment, trabeculectomy/drainage implant for glaucoma, any operation for rhegmatogenous retinal detachment (RRD) repair, pars plana vitrectomy (PPV) for vitreous hemorrhage (VH), macular hole, or retained lens fragments; elective procedures: corneal transplant, phacoemulsification for cataract extraction, silicone oil removal, and PPV for epiretinal membrane; and intravitreal injections (either anti-vascular endothelial growth factor [VEGF] or dexamethasone) to treat exudative maculopathies. The main outcome measure was the rate of reduction in urgent and elective surgeries performed.

**Results:**

Overall, 3624 and 844 surgical procedures were performed from 10 March to 9 April 2019 and from 10 March to 10 April 2020, respectively (−76.7% in 2020 compared to 2019, *p* < 0.0001). Urgent and elective surgical procedures and intravitreal injections of anti-VEGF drugs or dexamethasone reduced significantly in 2020 in comparison to 2019 (*p* < 0.0001 for urgent and elective surgeries and 0.01 for intravitreal injections).

**Conclusion:**

A significant reduction in the rate of urgent and elective surgeries and intravitreal injections was recorded during the first month of lockdown compared to the same period in 2019. With this analysis, the authors hope to provide some preliminary insights about the consequences of lockdown for the eyecare system in Italy.

## Introduction

On 10 March 2020, the Italian government implemented extraordinary measures to limit the spread of severe acute respiratory syndrome caused by coronavirus 2 (SARS-Cov-2).^1^ This has caused substantial changes to business, social, and sanitary practices including limitations in the access to hospitals and operating rooms for eyecare.

Such limitations, along with the patients’ fear or difficulties to get to hospitals, have resulted in a drastic reduction of eye surgical procedures carried out. Achieving a balance between infection control and the provision of ophthalmology services is crucial. In fact, many eye surgical treatment may be deferred, but some pathologies such as glaucoma, wet age-related macular degeneration, and RRD cause irreversible loss of visual function if treatment is not delivered in a timely manner.

The aim of this research is to provide an overview of the eye surgical procedures that were performed at six referral Institutional Ophthalmology Departments in Italy, one of the European countries most severely hit by the epidemic, during the first month of lockdown and to compare the number of procedures that were performed in this period with those performed during the same period last year. With this analysis, the authors hope to provide some preliminary insights about the consequences of lockdown for the eyecare system in Italy.

## Methods

Surgical records from 10 March to 9 April 2019 and 10 March to 9 April 2020 at six institutional centers that are located in the North (Department of Neurological and Vision Science, University of Brescia-Spedali Civili, Brescia; Department of Biomedical Science, Humanitas University, Bergamo; and Department of Morphology, Surgery and Experimental Medicine, University of Ferrara-Sant’Anna Hospital), Centre (Department of Ophthalmology, University of Florence-Careggi Hospital, Florence; and Department of Medicine and Health Sciences, University of Molise-Cardarelli Hospital, Campobasso), and South (Department of Ophthalmology University of Catania-Vittorio Emanuele General Hospital, Catania) of Italy were retrospectively reviewed.

During the period of lockdown, in order to achieve continuity of care and to limit the risk of contamination for both healthcare workers and patients, a series of control measures were adopted in the six centers involved in this study. Patients attending emergency department were screened for history of fever or upper respiratory symptoms, for domicile in one of the “red zones” and for contact with suspected or confirmed cases of COVID-19. Patients scheduled for elective surgery or intravitreal injections were contacted and screened by telephone according to the same protocol and asked to attend the visit with no more than one accompanying person in order to reduce the risk of exposure to subjects potentially infected by SARS-Cov-2. Whatever the surgical procedure to be performed (either urgent/elective surgery or intravitreal injection), the result of nasopharyngeal swab was mandatory before patients had access to operating rooms.

Surgical procedures were divided in urgent and elective according to recommendations that largely reflected those released by the American Academy of Ophthalmology.^2^ In general, a procedure was considered urgent when substantial deterioration of the patient’s eye condition and/or reduced recovery potential could likely occur if the surgery was delayed for ⩾10 days. Urgent surgeries included any trauma repairment (including suture of eyelids/cornea/sclera, traumatic cataract extraction and pars plana vitrectomy [PPV] for retinal detachment/removal of foreign bodies), trabeculectomy/drainage implant for glaucoma with intraocular pressure (IOP) uncontrolled on maximal medical therapy, scleral buckle/PPV to repair rhegmatogenous retinal detachment (RRD), PPV for acute vitreous hemorrhage (VH), recent-onset macular hole, or retained lens fragments causing inflammation. Elective surgeries included corneal transplant, phacoemulsification for cataract extraction, silicone oil removal, and PPV for epiretinal membrane. Furthermore intravitreal injections (either anti-vascular endothelial growth factor [VEGF] or dexamethasone) to treat exudative maculopathies (divided in those secondary to choroidal neovascularization [CNV], diabetic-related macular edema (DME) or retinal vein occlusion (RVO)-related macular edema) were analyzed.

The primary outcome was the comparison between the overall rate of surgical procedures performed in 2020 versus 2019. We calculated the incidence rates for the primary outcome by dividing the number of cumulative surgical procedures by the number of days for each time period (31 days). All subjects were treated in accordance with the Declaration of Helsinki. This study was approved by the Institutional Review Boards (IRBs) at the six institutional centers. Given the retrospective nature of the study, the IRBs waived the need of informed consent from participants.

Student *t*-test and chi-square test were used for comparisons of continuos and categorical variables. A value of *p* < 0.05 was considered significant. Statistical analysis was performed using MedCalc version 11.5.1 (MedCalc software, Mariakerke, Belgium).

## Results

The mean age (±standard deviation) of the patients who underwent surgery from 10 March to 9 April was 71.4 (±15.3) and 62.8 (±13.5) in 2019 and 2020, respectively (*p* = 0.009).

In all six centers, during the month of lockdown, eye surgical procedures were limited by reduced availability of operating rooms or shortage of personnel who was preferentially employed in intensive care units.

Overall, 3624 and 844 surgical procedures were performed from 10 March to 9 April 2019 and 10 March to 9 April 2020, respectively (−76.7% in 2020 compared to 2019, *p* < 0.0001). Only one center, Bergamo, located at the very epicenter of the epidemic in Italy, suspended every surgical procedure. In the other centers the mean reduction was 68.7%. Florence and Ferrara respectively were the centers with the most relevant and minimal reduction of surgeries performed in 2020 in comparison to 2019 (−85.4% and −34.7%, respectively). Three centers (Bergamo, Catania, and Campobasso) did not perform any elective surgery during the lockdown, two (Florence and Ferrara) reduced elective surgeries by more than 95% and one (Brescia) by more than 85%. Overall, the most relevant reduction was for cataract surgeries, which decreased from 1674 in 2019 to 37 in 2020 (−97.8%, *p* = 0.004). Cataract surgeries were performed exclusively for patients with best-corrected visual acuity (BCVA) in the fellow eye ⩽1.0 on the logarithm of the minimum angle of resolution scale. Among elective surgical procedures for vitreoretinal pathologies, PPV for ERM and PPV for silicone oil removal reduced by −93.9% and −93.6% (*p* = 0.007 and *p* = 0.02, respectively). As for cataract operations, only patients with BCVA ⩽ 1 logMAR in the fellow eye, underwent PPV for ERM during lockdown period. Silicone oil was removed in three cases presenting with substantial emulsification in the anterior segment and concomitant elevated IOP.

Regarding urgent operations, in Lombardy region, Bergamo stopped any surgery whereas a reduction of 57.7% was recorded in Brescia. For the remaining centers, the lowest reduction was recorded in Campobasso (−36%) and the highest in Catania (−77.4%). The other two centers (Ferrara and Firenze) had a quite similar reduction (−72% and −59.7%, respectively). Among urgent surgeries, the one that had the lowest reduction was drainage valve implantation (−28.6%, *p* = 0.56), but, overall, only 14 implants were performed in 2019. The other most common surgical procedure for glaucoma, that is, trabeculectomy, showed a more substantial reduction (−73.7%, *p* = 0.06). Urgent vitreoretinal surgical procedures such as RRD repair substantially, but to a minor extent, decreased in 2020 compared with 2019 (−64.2%, *p* = 0.04 globally and −83.7% and −54.6% for scleral encirclement/buckle and PPV, respectively, *p* = 0.07 for both procedures).

Trauma cases requiring surgery decreased from 17 in 2019 to 11 in 2020, reducing by 35.5%. Since there was no limitation to treat emergency cases in any of the centers surveyed, with the exception of Bergamo, it is possible that the reduction of trauma cases recorded during the first month of lockdown, was likely secondary to outdoor activities restrictions imposed by the government.

**Figure 1.**
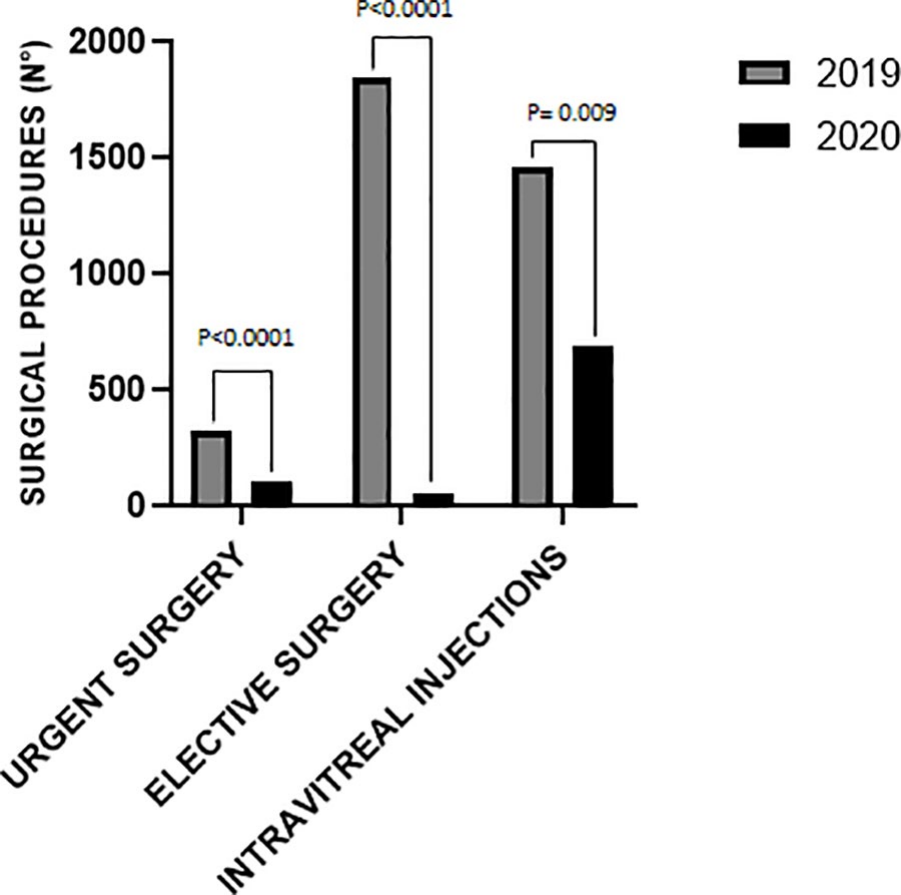
Comparison of the number of urgent/elective eye surgical procedures and intravitreal injections performed in the period 10 March to 9 April in 2019 and 2020 at six institutional centers in Italy.

**Figure 2.**
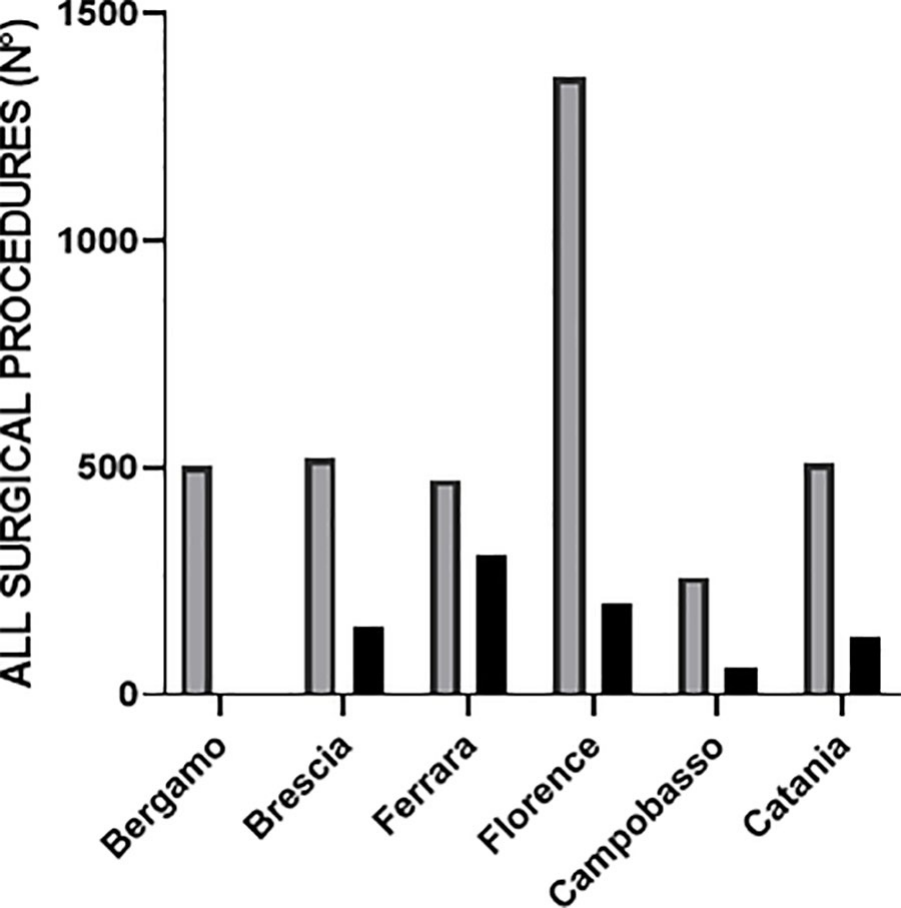
Comparison of the number of eye surgical procedures performed in the period 10 March to 9 April in 2019 and 2020 in each of the six Italian institutional centers surveyed.

**Figure 3.**
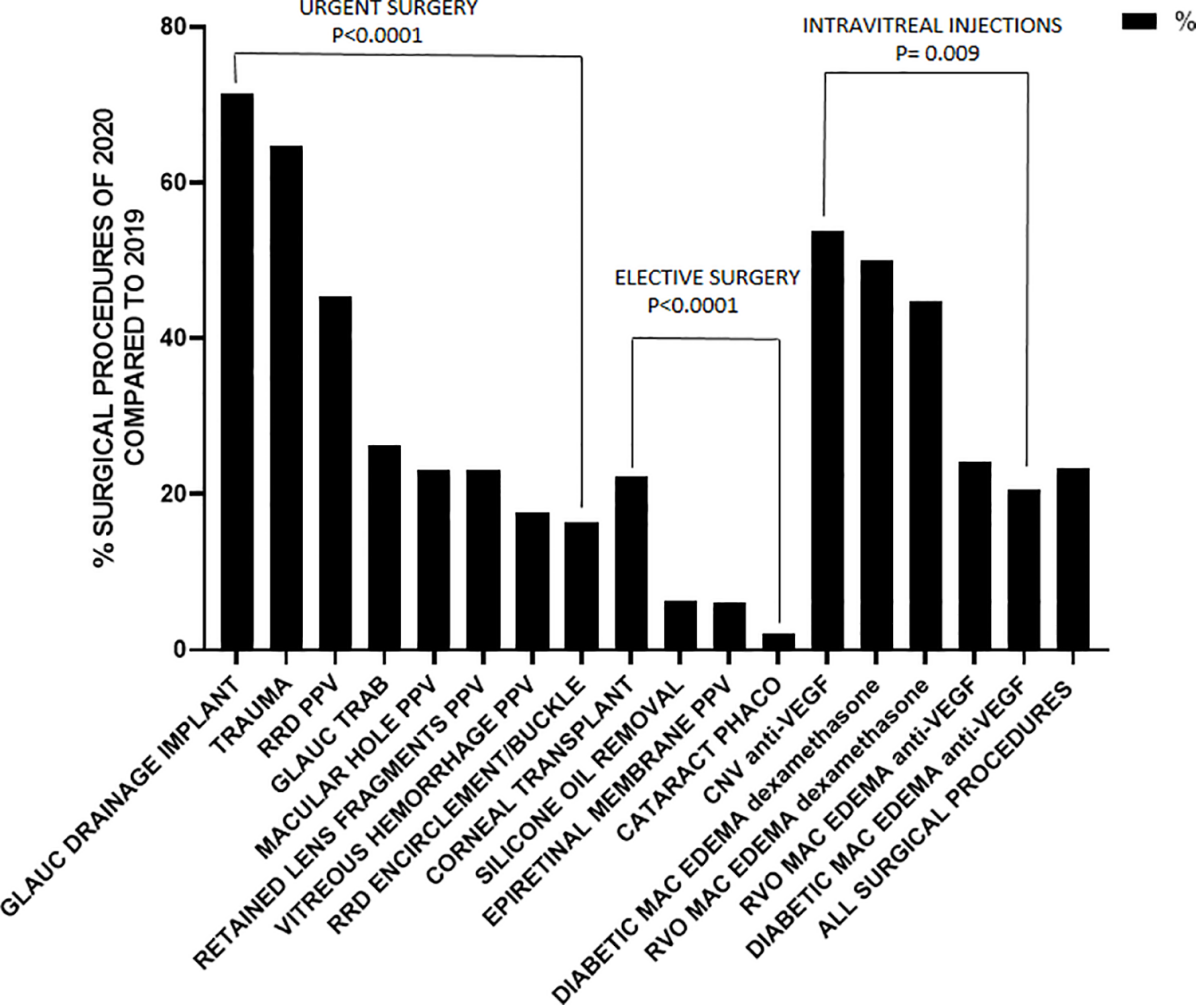
Percentage of elective and urgent surgical procedures performed in the period 10 March to 9 April 2020 (first month of lockdown in Italy because of COVID-19 epidemic) in comparison to the same period on 2019.

Finally, regarding intravitreal injections, the reduction was from 56% (Brescia and Catania) to 100% (Bergamo); in two centers injections reduced by slightly more than 70% (Florence and Campobasso), whereas in Ferrara increased by 17%. Globally, 702 fewer injections of anti-VEGF drugs and 70 fewer injections of dexamethasone were performed during the first month of lockdown compared to the same period in 2019, a reduction >50% (*p* = 0.08 and *p* = 0.01, respectively). When stratifying by pathology (CNV/DME/RVO-related macular edema), the reduction of anti-VEGF injections was more relevant for those performed for macular edemas than for CNV (−79.5%, −75.7%, and −46.1%, *p* = 0.006, *p* = 0.01, and *p* = 0.14 for DME, RVO-related macular edema, and CNV, respectively). (Table 1 and Figures 1–3).

**Table 1.** Comparison between ophthalmology surgeries performed in the period 10 March to 9 April 2020 (first month of national lockdown in Italy) with those performed in the period 10 March to 9 April 2019.

Pathology		Surgical procedure	No. of procedures	No. of procedures	No. of daily procedures	No. of daily procedures	Procedures	*p* Value^a^
			2019	2020	2019	2020	2020 versus 2019 (%)	
All pathologies		All surgical procedures	3624	844	116.9	27.2	−76.7	<0.0001*
Urgent surgery		Trauma, PPV or scleral buckling for RRD, PPV for VH, PPV for MH, PPV for retained lens fragments, trabeculectomy, GDI	320	106	9.9	3.4	−66.9	<0.0001*
Elective surgery		Corneal transplant, phacoemulsification, silicone oil removal, PPV for ERM	1846	52	59.5	1.7	−97.2	<0.0001*
Trauma		All trauma repairment procedures	17	11	0.5	0.3	−35.3	0.26
Corneal transplant		Any type of keratoplasty	27	6	0.9	0.2	−77.8	0.39
Cataract		Phacoemulsification	1674	37	54	1.2	−97.8	0.004*
Glaucoma		Trabeculectomy	38	10	1.2	0.3	−73.7	0.06
		Glaucoma drainage implant	14	10	0.4	0.3	−28.6	0.56
Vitreoretinal	Rhegmatogenous retinal detachment	All operations for RRD	148	53	4.8	1.7	−64.2	0.04
		Encirclement/buckle	49	8	1.6	0.2	−83.7	0.07
		Pars plana vitrectomy	99	45	3.2	1.4	−54.6	0.07
	Silicone oil in the vitreous cavity	Silicone oil removal	47	3	1.5	0.1	−93.6	0.02*
	Vitreous hemorrhage	Pars plana vitrectomy	34	6	1.1	0.2	−82.4	0.02*
	Epiretinal membrane	Pars plana vitrectomy	98	6	3.2	0.2	−93.9	0.007*
	Macular hole	Pars plana vitrectomy	43	10	1.4	0.3	−76.8	0.09
	Lens fragments retained in the vitreous	Pars plana vitrectomy	26	6	0.8	0.2	−77	0.08
Exudative maculopathies	All	Anti-VEGF intravitreal injections	1322	620	42.6	20	−53.1	0.008*
	Choroidal neovascularization		1034	557	33.3	18	−46.1	0.14
	Macular edema (diabetes)		185	38	6	1.2	−79.5	0.006*
	Macular edema (vein occlusion)		103	25	3.3	0.8	−75.7	0.01*
Exudative maculopathies	All	Dexamethasone intravitreal injections	136	66	4.4	2.1	−51.5	0.01*
	Macular edema (diabetes)		98	49	3.2	1.6	−50	0.05
	Macular edema (vein occlusion)		38	17	1.2	0.5	−55.3	0.05

PPV: pars plana vitrectomy; RRD: rhegmatogenous retinal detachment; VH: vitreous hemorrhage; MH: macular hole; GDI: glaucoma drainage implant; ERM: epiretinal membrane; VEGF: vascular endothelial growth factor.

a Chi-square test.

* Statistically significant.

## Discussion

From 10 March 2020, the beginning of the lockdown on national scale that was set by the government in Italy because of the spread of the COVID-19 epidemic, people across the country were asked to not leave their homes unless strictly necessary. Travel between different municipalities was banned except for emergencies, work, or health reasons. Commercial businesses limited the number of individuals in the store, and health care settings drastically limited or even abolished scheduled visits or operations.

The Italian health system’s capacity to respond to these changing circumstances has been under enormous pressure, mostly in Lombardy, the region in Italy and one of the areas in the world most severely hit by the epidemic.^3^

In an attempt to analyze the repercussions of the containment measures on the eyecare system, we compared the more common surgical procedures that were performed at six institutional centers located in North, Central, and South Italy during the first month of lockdown with those performed during the same period in 2019.

The mean age of the patients who underwent surgical procedures was significantly lower in the month of lockdown compared with the same period in 2019. The need to prioritize urgent treatment and the consequent cancellation of elective surgeries along with the difficulties encountered by older people, more vulnerable to COVID-19 infection, in getting to the hospitals or their fear of contracting the disease in nosocomial environments, may explain these results.

The reduction of all surgical procedures performed was globally >70% and quite homogeneous in the centers under consideration with the exceptions of Bergamo which had a 100% reduction and Ferrara which had a reduction of only 34.7%. In Bergamo, located at the very epicenter of the epidemic in Italy, eye surgical procedures were completely stopped in order to diverge resources to intensive care units. The figures relating to Ferrara can be explained by the increased number of intravitreal injections performed in this center during the month of lockdown in comparison to the same month of 2019, that balanced the reduction of elective and urgent procedures. It must be said that all centers tried to maintain intravitreal injections administration for patients with CNV and also for patients with macular edema secondary to diabetes (DME) and retinal vein occlusion in cases presenting with significant vision loss, or for monocular or quasi-monocular patients (only one eye >20/40). This was in line with the recommendations about intravitreal injections administration during pandemic released by international committees.^2,4^

Depending on several factors like the availability of resources, the number of COVID-19+ patients in each region and the volume of operations carried out under normal circumstances, differences in terms of reduction of eye surgeries and intravitreal injections were found among the six centers.

For instance, according to the data, it appeares that Ferrara, differently from the other centers, managed to perform the majority of scheduled and new intravitreal injections. On the other hand, it was the center with the second highest reduction of elective and urgent surgeries (−95.5% and −72%, respectively). Florence was the center with the highest percent reduction of surgical procedures in comparison to 2019 (−85.4%) but the absolute numbers of elective/urgent surgeries and intravitreal injections performed during the month of lockdown were the highest among the other centers.

The figures from Ferrara and Florence are thus emblematic and very helpful to understand how differences in the reorganization of the eye services and availability of staff and supplies may have had different effects on the number and type of procedures performed in every single center. Nevertheless, as common denominator, in all centers there was a general reduction in the number of the surgical procedures performed, with the most relevant decrease in elective, scheduled surgeries such as cataract operations.

With a global prevalence that is almost 50% in adults who are over 50 years of age, cataract surgery is the most common surgical procedure that is performed in developed countries.^5^ In Italy, more than 6,000,000 cataract surgeries are performed each year. In an Italian population-based survey, cataracts at advanced stages were found in about two-thirds of subjects aged 70 years or more with a decrease in visual acuity to under 0.7 in the worst eye found in 54.4% of them.^6^ Thus, cataract has a high prevalence in elderly Italian people and plays an important role on the quality of life of patients aged ⩾70 years.

In the first month of lockdown, only 37 phacoemulsification procedures were performed at the six centers involved in this study, compared to the 1674 surgeries that were performed during the same period in 2019 (−97.8%). This means that, on national scale, 1 month of lockdown may have caused a reduction of more than 50,000 cataract surgeries compared to 2019.

However, even the number of procedures requiring more prompt intervention such as those to treat glaucoma or repair RRD substantially decreased. For instance, the number of trabeculectomies for glaucoma, was substantially reduced from 38 procedures in 2020 to 10 in 2019 (−73.7%) This is important because patients with uncontrolled IOP on maximal medical treatment, requiring incisional glaucoma surgery, may lose their vision in a matter of few weeks.

Data for RRD treatment seem to be are even more alarming. Surgeries for RRD repair dropped from 148 in 2019 to 53 in 2020 (−64.2%). Although, an analysis of the time between diagnosis and surgery was not performed in this study, it is likely that, given a similar incidence of RRDs in Italy in 2019 and 2020, almost two-thirds of RRD repair surgeries were delayed during this month of lockdown.

This delay may have had relevant consequences on visual recovery since many studies have shown that poorer visual outcomes are associated with longer durations of preoperative detachment, independently of the surgical procedures (scleral buckling or vitrectomy) that are chosen to repair the RRD.^7–9^

For intravitreal injections, an overall decrease of >50% in the number of injections that were administered was recorded for both anti-VEGF drugs and dexamethasone in 2020 compared to the same period of 2019. For anti-VEGF injections, the decrease was higher for those that were administered for diabetes- and RVO-related edema compared to CNV (−78.1% vs −46.2%). This likely reflects the decision of clinicians to prioritize the treatment of wet age-related macular degeneration ( the main cause of CNV), a condition that may cause a more rapid vision loss compared to long-standing macular edema that is related to diabetes or RVO. However, according to our data, the injections that were administered for CNV were almost halved in the first month of lockdown compared to the same period in 2019. Should the inability to adhere to more strict treatment protocols last for several months more, this might lead to very poor visual outcomes in the near future for these patients who are left undertreated because of the restrictions that are imposed by the COVID-19 outbreak.^10–12^

There are several limitations in this study. First, it is a retrospective study with limitations that are inherent to such a study design. Second, the number of patients analyzed is relatively small. Third, although data were gathered from six institutional centers located in five different regions of Italy, it is possible that they do not exactly reflect the overall national reduction in treated ophthalmology cases. Fourth, the conclusions of this study may not be completely applicable to other countries in the world in which restrictions imposed during lockdown period may have been somewhat different.

Nevertheless, since the containment measures taken in most European countries were similar to those taken in Italy, we believe that the insights from this study could serve to inform physicians, administrators, and policy makers about what COVID-19 epidemic caused to and may cause to eyecare in the future, should Italy and Europe experience a second wave of infection. In Italy, phase two, which is the intermediate period after the stringent lockdown measures, started on 4 May 2020. The slow return to normality will be inevitably influenced by the development of effective therapies and safe vaccines and a better understanding of the immunity gained by people who have recovered from infection. In addition, we believe that the knowledge of the effects caused by the epidemic on standard eyecare will be of paramount importance in order to better planning more effective sanitary responses in the event of COVID-19 resurgence.
